# Knowledge, perception, and willingness to provide telepharmacy services among pharmacy students: a multicenter cross-sectional study in Indonesia

**DOI:** 10.1186/s12909-023-04790-4

**Published:** 2023-10-26

**Authors:** Sofa D. Alfian, Qisty A. Khoiry, Mochammad Andhika A. Pratama, Ivan S. Pradipta, Susi A. Kristina, Elida Zairina, Eelko Hak, Rizky Abdulah

**Affiliations:** 1 Department of Pharmacology and Clinical Pharmacy, Faculty of Pharmacy, Universitas Padjadjaran, Jatinangor, Indonesia; 2 Drug Utilization and Pharmacoepidemiology Research Group, Centre of Excellence for Pharmaceutical Care Innovation, Universitas Padjadjaran, Jatinangor, Indonesia; 3 Center for Health Technology Assessment, Universitas Padjadjaran, Jatinangor, Indonesia; 4 Department of Pharmaceutics, Faculty of Pharmacy, Universitas Gadjah Mada, Yogyakarta, Indonesia; 5 Department of Pharmacy Practice, Faculty of Pharmacy, Universitas Airlangga, Surabaya, Indonesia; 6 Innovative Pharmacy Practice and Integrated Outcome Research (INACORE) Group, Universitas Airlangga, Surabaya, Indonesia; 7 Center of Excellence for Patient Safety and Quality, Universitas Airlangga, Surabaya, Indonesia; 8 Unit of PharmacoTherapy, -Epidemiology, & -Economics, Groningen Research Institute of Pharmacy, University of Groningen, Groningen, the Netherlands

**Keywords:** Knowledge, Perception, Willingness, Telepharmacy, Students, Indonesia

## Abstract

**Introduction:**

The coronavirus disease 2019 (COVID-19) pandemic accelerated the provision of telepharmacy services. However, little is known about the knowledge, perception, and willingness of pharmacy students as future key players in telepharmacy adoption to provide such a service, particularly in a setting without well-established telepharmacy services before the COVID-19 pandemic.

**Objective:**

With this survey we aimed to assess the level of knowledge, perception, and willingness to provide telepharmacy services and to identify associated factors among pharmacy students in Indonesia.

**Methods:**

We applied a multicenter cross-sectional study design with convenience sampling technique among pharmacy students in three public universities in Bandung City, Surabaya City, and Special Region of Yogyakarta, Indonesia. The knowledge, perception, and willingness to provide telepharmacy services were assessed using an online questionnaire. Ordinal regression analysis was performed to determine factors associated with a high knowledge level, whereas binary logistic regression analyses were performed to determine factors associated with a positive perception of telepharmacy services. Odds ratios (ORs) with 95% confidence intervals (CIs) were reported.

**Results:**

Among 313 respondents, 83.4% were female, and the mean age was 20 years. Although only 13.2% showed a high knowledge level, 66.5% showed a positive perception of telepharmacy services and 97.4% were willing to provide telepharmacy services in the future. An increase in age (OR 1.33; 95% CI 1.14–1.54) and being advance in smartphone usage (OR 5.21; 95% CI 2.03–13.42) are associated with an increased likelihood of having a high knowledge level about telepharmacy services. Male students had a lower likelihood of having a positive perception of telepharmacy services than females (OR 0.46; 95% CI 0.24–0.85).

**Conclusion:**

Despite limited knowledge of telepharmacy, the majority of pharmacy students reported a positive perception and willingness to provide telepharmacy services in their future careers. Therefore, telepharmacy practice models must be included as a subject course in the curriculum, better preparing future pharmacists to perform their roles effectively. Furthermore, student-specific factors such as age and expertise in smartphone usage that associated with knowledge and gender that associated with perception should be considered to facilitate telepharmacy adoption in Indonesia.

**Supplementary Information:**

The online version contains supplementary material available at 10.1186/s12909-023-04790-4.

## Introduction

The coronavirus disease 2019 [COVID-19] pandemic has significantly affected the provision of pharmaceutical care [1]. As several movement restrictions have been implemented, the number of patients attending outpatient care had significantly decreased [2]. Therefore, telepharmacy services, an approach for delivering pharmaceutical care through the use of digital health technologies which enabling pharmacists and patients to interact remotely and minimizing patients’ exposure to healthcare settings [3], are recommended as an adaptive method [4, 5]. These changes may not be temporary; thus, both pharmacists and pharmacy students must prepare themselves to provide telepharmacy services to improve the health and wellbeing of patients [6].

Previous studies have reported that outcomes provided by telepharmacy services are relatively similar to those of traditional pharmaceutical care services [7, 8] and can improve patient satisfaction [8–11]. However, the implementation of telepharmacy services requires a unique set of skills that are currently lacking in pharmacy education [11–15]. Therefore, pharmacists may experience difficulty in integrating clinical skills used for in-person counseling with the virtual environment [11] because existing skills and resources are often set up to support face-to-face counseling. Without sufficient telepharmacy training, pharmacy students were reported to be more successful at providing face-to-face consultation than via telepharmacy [16].

The adoption rate of telepharmacy was reported to be low in many countries [17], particularly in low- and middle-income countries without well-established telepharmacy services even before the COVID-19 pandemic. One of the facilitating factors for the successful adoption of telepharmacy is acceptance [18], by either current or future pharmacists. Therefore, early assessment of pharmacy students’ knowledge and perception is important to measure their acceptance of telepharmacy services. Adequate knowledge and perception can enhance their willingness to provide telepharmacy services in their future careers [19–21]. Such information is needed for developing and implementing capacity-building initiatives in pharmacy students. However, to date, no study has assessed the level of knowledge, attitude, and willingness of pharmacy students to provide telepharmacy services in any form in Indonesia, limiting evidence-based policy for its adoption. Therefore, we aimed to assess the level of knowledge, perception, and willingness to provide telepharmacy services and to identify factors associated with a high knowledge level and positive perception of telepharmacy among pharmacy students in Indonesia.

## Methods

### Study design, population, and setting

We applied a multicenter cross-sectional survey design among pharmacy students in three public universities in Bandung City, Surabaya City, and Special Region of Yogyakarta in Indonesia from August to September 2022. All pharmacy students of these universities were deemed eligible. Pharmacy students who did not live in Indonesia over the last 3 years were excluded.

The study was approved by the Health Research Ethics Committee of Universitas Padjadjaran, Indonesia (no. 614/UN6.KEP/EC/2022). A written informed consent was obtained electronically before proceeding with the survey. This study was reported according to the Strengthening the Reporting of Observational Studies in Epidemiology guidelines for cross-sectional studies [22] (Table S1, Supplementary data).

### Data collection

Students registered in a bachelor of pharmacy from all study levels (first to fourth years) were approached online by a convenience sampling technique. The link to the survey and study information outlining the brief description of the study were exclusively administered online using Qualtrics through individual and group chats with the assistance of their respective class representatives. The questionnaire could only be filled once from a single email address to avoid duplicate entries. To ensure anonymity, the names and email addresses of the students were not recorded. The survey took an average of 10 min to complete. The questionnaire consisted of four sections, namely, demographic characteristics, knowledge, perception, and willingness to provide telepharmacy services. Questions about knowledge, perception, and willingness to provide telepharmacy services were based on a previously published study [23]. We followed the guidelines developed by The International Society for Pharmacoeconomics and Outcome Research (ISPOR) for the adaptation process of the questionnaires [24]. The questionnaire was translated into Indonesian (forward translation) and retranslated into English (back translation) by two independent professional language translators. The translated version of the questionnaire was shared with three experts in clinical pharmacy from pharmacy academicians. Subsequently, expert opinion regarding the clarity of each item was considered before finalizing the questionnaire. Following content and face validity, a pilot study was conducted on 34 pharmacy students to verify the comprehensibility of the questionnaire. As a consequence, we modified the original version of the questionnaire based on feedback and cross-validation analysis, particularly on the questions to assess willingness of pharmacy students to provide telepharmacy services. Out of the original six willingness questions, only one question was utilized in this study due to the presence of multicollinearity. Furthermore, we have removed a question related to the willingness to pay for the telepharmacy application. This question was deemed not applicable to our target population of students, and its inclusion would have introduced confusion and potential bias to the study results.

The Indonesian version of the questionnaire showed to be valid (the correlation values of each question to the total score were > 0.679, > 0.645, and > 0.346 for knowledge, perception, and willingness to provide telepharmacy services, respectively) and reliable (Cronbach alpha coefficients of 0.782, 0.797, and 0.651 for knowledge, perception, and willingness to provide telepharmacy services, respectively).

### Demographic characteristics

This section explored the diversity of students, which may be associated with their knowledge, perception, and willingness to provide telepharmacy services using questions on age on completion of the questionnaire, sex (male and female), and grade point average (GPA). Factors related to their experience in using a smartphone were also explored, such as perceived expertise in smartphone usage (beginner, expert, or advance), duration of smartphone use in a day (1–4 or ≥ 5 h), and availability of internet access at home (yes or no).

### Knowledge of telepharmacy services

Knowledge of telepharmacy services was assessed using the following five questions: “To what extent are you familiar with the benefits of telepharmacy?,” “To what extent are you familiar with telepharmacy applications and platforms in Indonesia?,” “To what extent are you familiar with the use of telepharmacy abroad?,” “To what extent are you familiar with recently released guidelines of telepharmacy in Indonesia?,” and “To what extent do you participate in conferences and webinars related to telepharmacy?” [23]. Items in this section were rated based on a 5-point Likert scale ranging from “1 = very low” to “5 = very high.” We categorized the knowledge total score based on the Bloom cut-off point as follows: students with a total score between 20 (80%) and 25 (100%) were categorized as having a high knowledge level; a total score between 15 (60%) and 19.75 (79%), moderate knowledge; and a total score of < 15 (< 60%), low knowledge [25, 26].

### Perception of telepharmacy services

Perception of telepharmacy services was assessed by the following five statements: “Telepharmacy is a viable approach for providing comprehensive healthcare to the patient,” “Telepharmacy enables the adoption of technology in healthcare,” “Telepharmacy saves time and reduces effort (workload),” “Telepharmacy helps in reducing the cost of service,” and “There are existing telepharmacy applications in Indonesia that can be easily adopted” [23]. Items in this section were classified using dichotomous scores: “agree” (1 point) and “disagree” (0 points). The total score of < 2.5 (50%) was categorized as a negative perception, whereas a total score of ≥2.5 (50%) was categorized as a positive perception of telepharmacy services [23].

### Willingness to provide telepharmacy services

This section assessed the willingness of students to provide telepharmacy services in their future careers based on this question: “Are you willing to integrate telepharmacy with the existing system to provide care in the future?” [23]. Items in this section were classified using dichotomous scores: “agree” (1 point) and “disagree” (0 points).

### Sample size calculation

The sample size was calculated using Slovin’s formula [27] because no prior knowledge on outcomes distributions is available. To obtain a 95% confidence interval (CI) and a margin error of 0.10 based on a statistical power of 80%, a minimum sample size of 300 pharmacy students was needed.

### Data analysis

Descriptive statistics were used to summarize the characteristics of the pharmacy students. Multicollinearity was analyzed before entering variables into the multivariate regression analysis using the variance inflation factor. Two regression models were used, each for knowledge and perception of telepharmacy services. Ordinal regression analysis was conducted to assess univariate and multivariate associations of pharmacy students’ characteristics with their knowledge of telepharmacy services. Binary logistic regression analysis was conducted to assess univariate and multivariate associations of pharmacy students’ characteristics with their perception of telepharmacy services. The odds ratio (OR) and 95% confidence interval (CI) were reported. A p-value of < 0.05 was considered statistically significant. All statistical analyses were performed using IBM SPSS Statistics for Windows version 27.0 (IBM Corp., Armonk, NY, USA).

## Results

### Baseline characteristics

A total of 313 pharmacy students were included in this study, 83.4% were female (mean age, 20 years), 76.7% were perceived as expert in smartphone usage, and 78.0% used smartphones for > 5 h a day (Table 1). Although only 13.2% showed a high knowledge level, 66.5% showed a positive perception of telepharmacy services and 97.4% were willing to provide telepharmacy services in the future (Table 1).

**Table 1:** Characteristics of the respondents (N = 313)

Characteristics	n (%)
**Sex**	
Male	52 (16.6)
**Age in years, mean (SD)**	20.0 (1.63)
GPA scale*, mean (SD)	3.4 (0.11)
**Self-reported expertise in smartphone usage**	
Beginner	38 (12.1)
Expert	240 (76.7)
Advance	35 (11.2)
**Duration of smartphone use in a day**	
1–4 h	69 (22.0)
≥ 5 h	244 (78.0)
**Availability of Internet access at home**	
Yes	266 (85.0)
No	47 (15.0)
**Knowledge of telepharmacy services**	
Low	83 (26.5)
Moderate	189 (60.4)
High	41 (13.1)
**Perception of telepharmacy services**	
Positive	208 (66.5)
Negative	105 (33.5)
**Willingness to provide telepharmacy services in the future**	
Yes	305 (97.4)
No	8 (2.6)

*Note: GPA, grade point average, the average value of the accumulated final grades earned in courses over time, range: 1.0–4.0

**Fig. 1:**
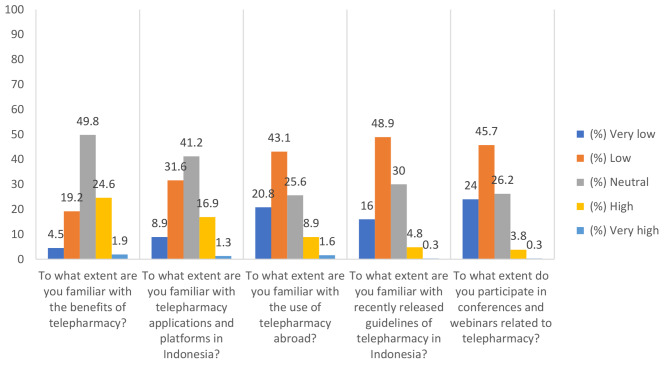
Responses to knowledge about telepharmacy services (N = 313)

Among pharmacy students with knowledge of telepharmacy services, 24.6% were highly and 1.9% were very highly familiar with the benefits of telepharmacy, however 31.6% have low and 8.9% have very low familiarity with its applications and platforms in Indonesia (Fig. 1). Moreover, 48.9% have low and 16.0% have very low familiarity with the recent released guidelines of telepharmacy in Indonesia and 45.7% have low and 24.0% have very low participation in any telepharmacy-related training (Fig. 1.).

**Fig. 2:**
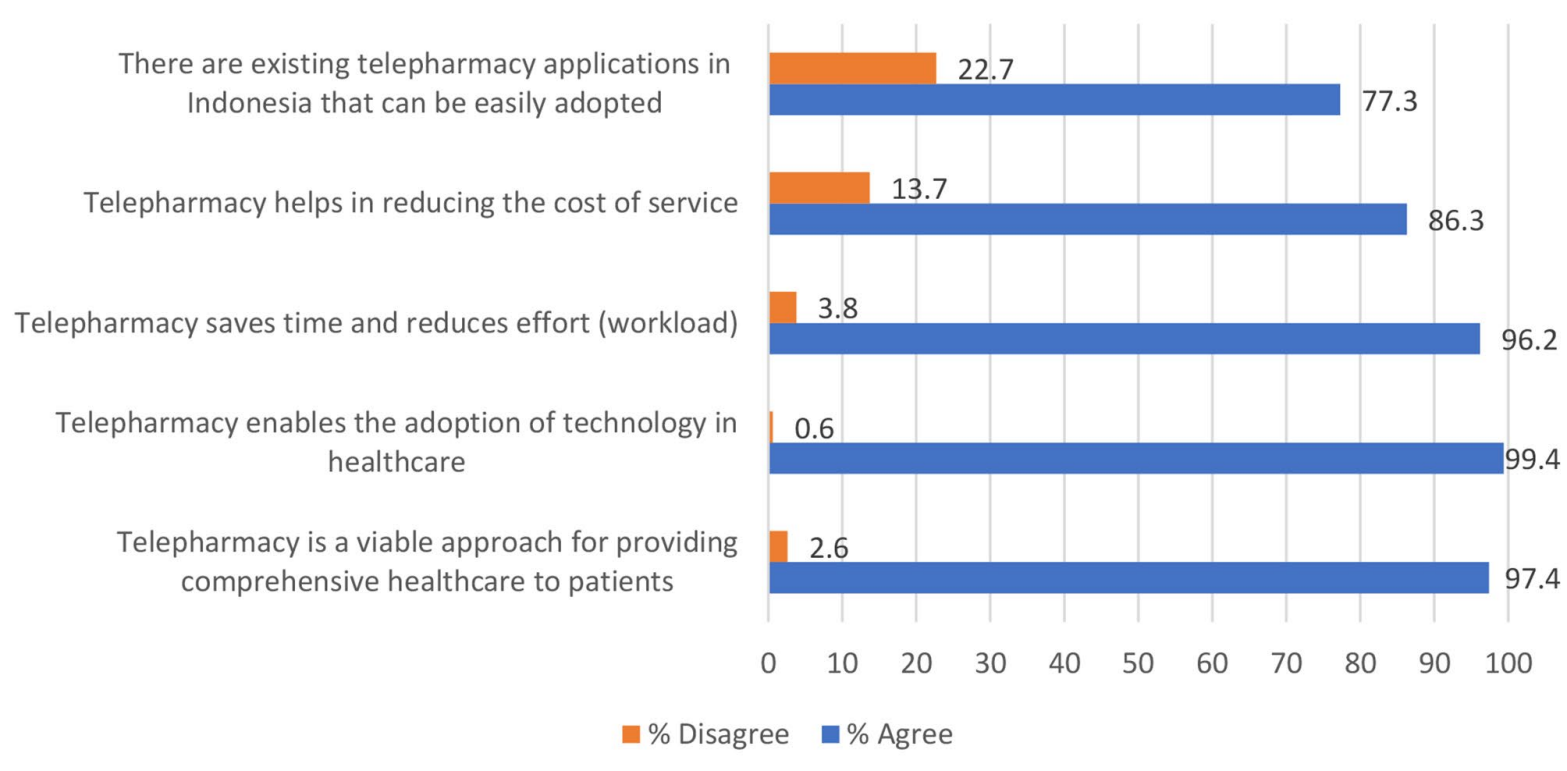
Responses to perception of telepharmacy services (N = 313)

Most of the pharmacy students perceived that telepharmacy offered several benefits such as the provision of comprehensive healthcare to the patient (97.4%), adoption of technology in healthcare (99.4%,), saving time and reducing effort (96.2%), and reduction of the cost of service (86.3%). However, 77.3% of them agree that there are existing telepharmacy applications in Indonesia that can be easily adopted (Fig. 2.).

### Factors associated with knowledge and perception of telepharmacy services

No significant multicollinearity was observed (variance inflation factors < 10). In the adjusted multivariate model, an increase in age (OR 1.33; 95% CI 1.14–1.54) and being an expert (OR 2.52; 95% CI 1.28–4.99) and advance (OR 5.21; 95% CI 2.03–13.42) in smartphone usage are associated with an increased likelihood of having a high knowledge level relative to moderate and low level of knowledge about telepharmacy services (Table 2). Meanwhile, male students (OR 0.46; 95% CI 0.24–0.85) are associated with a lower likelihood of having a positive perception of telepharmacy services (Table 3).

**Table 2:** Factors associated with a high knowledge level of telepharmacy services (N = 313)

Characteristics	Knowledge			Bivariate		Multivariate^†^	
	Low (n = 83)	Moderate (n = 189)	High (n = 41)	OR (95% CI)	p-value	OR (95% CI)	p-value
Sex, N (%)							
Female	64 (24.52)	166 (63.60)	31 (11.88)	Ref		Ref	
Male	19 (36.54)	23 (44.23)	10 (19.23)	0.81 (0.44–1.49)	0.493	0.77 (0.41–1.44)	0.415
Internet access at home, N (%)							
Yes	67 (25.19)	161 (60.53)	38 (14.29)	Ref		Ref	
No	16 (34.04)	28 (59.57)	3 (6.38)	0.59 (0.32–1.09)	0.093	0.65 (0.36–1.23)	0.188
Expertise in smartphone usage, N (%)							
Beginner	16 (42.11)	21 (55.26)	1 (2.63)	Ref		Ref	
Expert	63 (26.25)	145 (60.42)	32 (13.33)	2.29 (1.18–4.45)	0.015	2.52 (1.28–4.99)	0.008*
Advance	4 (11.43)	23 (65.71)	8 (22.86)	5.15 (2.05–12.92)	0.000	5.21 (2.03–13.42)	0.001*
Duration of smartphone use in a day, N (%)							
1–4 h	17 (24.64)	45 (65.22)	7 (10.14)	Ref		Ref	
≥5 h	66 (27.05)	144 (59.02)	34 (13.93)	1.02 (0.61–1.74)	0.921	0.91 (0.53–1.57)	0.744
Age, mean (SD)	19.45 (1.22)	20.14 (1.75)	20.59 (1.45)	1.33 (1.14–1.57)	0.000	1.33 (1.14–1.54)	0.000*
GPA scale, mean (SD)	3.42 (0.12)	3.41 (0.12)	3.44 (0.06)	1.28 (0.19–8.46)	0.796	1.37 (0.20–9.20)	0.747

Note: GPA, grade point average; OR, odds ratio

^†^pseudo R-squared: 5.71%

*p < 0.001 at the 5% level of significance

**Table 3:** Factors associated with a positive perception of telepharmacy services (N = 313)

Characteristics	Perception		Bivariate		Multivariate^†^		
	Negative (n = 105)	Positive (n = 208)	Odds ratio (95% CI)	p-value	Odds ratio (95% CI)	p-value	
Sex, N (%)							
Female	80 (30.65)	181 (69.35)	Ref		Ref		
Male	25 (48.08)	27 (51.92)	0.48 (0.26–0.87)	0.016	0.46 (0.24–0.85)	0.013*	
Internet access at home, N (%)							
Yes	86 (32.33)	180 (67.67)	Ref		Ref		
No	19 (40.43)	28 (59.57)	0.70 (0.37–1.33)	0.280	0.77 (0.40–1.49)	0.442	
Expertise in smartphone usage, N (%)							
Beginner	13 (34.21)	25 (65.79)	Ref		Ref		
Expert	84 (35.00)	156 (65.00)	0.96 (0.47–1.99)	0.925	0.90 (0.43–1.90)	0.791	
Advance	8 (22.86)	27 (77.14)	1.75 (0.61–5.02)	0.288	1.86 (0.64–5.44)	0.254	
Duration of smartphone use in a day, N (%)							
1–4 h	23 (33.33)	46 (66.67)	Ref		Ref		
≥5 h	82 (33.61)	162 (66.39)	0.99 (0.56–1.74)	0.966	0.96 (0.53–1.72)	0.884	
Age, mean (SD)	20.27 (1.43)	19.88 (1.7)	0.86 (0.74–1.01)	0.061	0.86 (0.73–1.01)	0.051	
GPA scale, mean (SD)	3.43 (0.1)	3.41 (0.1)	0.37 (0.04–3.35)	0.380	0.43 (0.04–4.52)	0.488	

Note: GPA, grade point average; OR, odds ratio

^†^pseudo R-squared: 4.93%

*p < 0.05 at the 5% level of significance

## Discussion

Although only a small number of pharmacy students in our study showed a high knowledge level, most of them showed a positive perception and willingness to provide telepharmacy services in the future. An increase in age is associated with an increased likelihood of having a high knowledge level of telepharmacy services. Being a male student is associated with a lower likelihood of having a positive perception of telepharmacy services.

We observed that the proportion of female pharmacy students (74.4%) is higher than male pharmacy students (16.6%) in this study which is in line with national data showing that females will represent around 86% of pharmacists in Indonesia by 2030 [28]. We further observed that the level of knowledge of telepharmacy among pharmacy students in this study is lower than those of pharmacy students in Malaysia [21] and the USA [29] in which majority of the respondents also female pharmacy students. While most of them recognized the benefits of telepharmacy services, more than half of them were not aware of telepharmacy applications and its recent guidelines in Indonesia. The reason is that telepharmacy applications have not yet been made widely available in Indonesia as compared with telemedicine [30]. The prominence and availability of telemedicine services have garnered more attention and resources compared to telepharmacy in Indonesia, which might explain the low level in knowledge among more than half of pharmacy students. Furthermore, this lack of knowledge of telepharmacy services (e.g., medication selection, order review and dispensing, patient counselling and monitoring) may be attributed to the lack of regulations, insufficient information dissemination, and inadequate marketing of telepharmacy platforms. Another reason for the unsatisfactory level of knowledge is the absence of telepharmacy training courses in the curricula. Only a few pharmacy students are educated regarding telepharmacy, which is understandable because of the low number of students having attended any telepharmacy-related training through conferences or webinars. Therefore, an approach to promoting the adoption of telepharmacy services is to expose pharmacy students to telepharmacy practices earlier during their education in bachelor degree [11, 31, 32] as it has a great influence on their knowledge and opinion regarding their future work [20]. The integration of telepharmacy services into training programs has been shown to improve students’ comprehension of drug therapy problems and their capacity to communicate patient care plans [33, 34].

Despite only a small number of pharmacy students showing a high knowledge level, the majority of them showed a positive perception and willingness to provide telepharmacy services in the future. This is in line with the results of previous studies conducted among pharmacy students in the USA [29] and medical and allied healthcare students in India [23]. In the present study, students’ perceptions of telepharmacy services appear favorable, i.e., they agreed that telepharmacy services would benefit them in terms of saving time and workload. However, they remain unaware of existing telepharmacy applications that can be easily adopted in Indonesia. Again, this might be due to insufficient information dissemination and inadequate marketing of telepharmacy platforms. Furthermore, a smaller percentage of students were skeptical about the possibility of telepharmacy in reducing the cost of service, particularly if telepharmacy devices or services were overpriced. This is an important factor for decision makers to make telepharmacy services affordable and accessible. Moreover, the introduction of mandatory telepharmacy courses in the curriculum may improve students’ perceptions. This was demonstrated by a study conducted among undergraduate students in Australia where a statistically significant difference in perceptions was found before and after the eHealth workshop [35].

Furthermore, nearly all pharmacy students in our study were willing to provide telepharmacy services in the future, which is an important prerequisite for the successful adoption of telepharmacy services. This could indicate that telepharmacy services have the potential to be well implemented in future pharmacy practice in Indonesia, particularly beyond the COVID-19 pandemic. However, other factors should be also considered in implementing telepharmacy, such as regulation, infrastructure, facility, and patient acceptability [36, 37].

An increase in age is associated with an increased likelihood of having a high knowledge level of telepharmacy services. Older age may have higher exposure to diverse materials of pharmacy curriculum–including telepharmacy, due to their higher semester level. However, it is important to consider that a significant number of them still have limited engagement in telepharmacy-related activities in this study. Furthermore, being an expert and advance in smartphone usage are associated with an increased likelihood of having a high knowledge level of telepharmacy services compared to a beginner. The higher self-reported expertise in smartphone usage may be indicating the higher eHealth literacy [38] which may l﻿ead to their high knowledge level of telepharmacy services. Meanwhile, male students are associated with a lower likelihood of having a positive perception of telepharmacy services. This is surprising because men were previously reported to have a greater affinity for technology than women, which is reflected by a higher adoption of eHealth and telemedicine [39]. On the other side, men may have a negative perception of telepharmacy due to their concerns regarding the intricacies and technical aspects of the technology involved such as security concern, as they have greater affinity for technology [39]. The use of technology and remote communication in telepharmacy can lead to concerns about the security and privacy of personal health information, as well as doubts about the confidentiality and reliability of telepharmacy services [40]. Several strategies can be employed to promote a more positive perception, such as emphasize the robust security measures in place to protect patient information and ensure confidentiality, highlight the encryption protocols, secure platforms, and establish regulatory frameworks that govern the development and management health information technology systems [41–43].

In this study, no association was found between internet access at home, expertise in smartphone usage, and duration of smartphone use toward knowledge about telepharmacy services. This may be due to digitalization where everyone has access to the internet regardless of smartphone-related factors.

To the best of our knowledge, this is the first study conducted in Indonesia to assess the knowledge, perception, and willingness to provide telepharmacy services in a higher education setting. Furthermore, this study was conducted in different universities in different regions of Indonesia, which strengthens the generalizability of the results. However, some limitations should be acknowledged. Data collection using an online questionnaire has several methodological drawbacks: the lack of a trained interviewer to clarify the information provided can lead to less reliable data, and voluntary participation can result in participants with biases selecting themselves into the sample. However, the characteristics of pharmacy students in this study are comparable with other studies conducted among pharmacy students in Indonesia [44, 45]. We could not calculate the response rate because of the lack of data regarding the number of potentially eligible students who refused to participate in this study. However, as expected, the response rates in online surveys were lower than those in paper-based surveys [46]. We also could not draw causal inferences regarding the temporal associations between potential factors and outcomes. Furthermore, as with any survey, the data used were self-reported; thus, this study is prone to desirability bias. Although this study achieved the estimated sample size, we believe that a large sample from more cities in Indonesia may provide slightly different results. A stepwise approach was used to determine the variables that should be included in the model, which may be prone to chance findings. Future studies should justify the choice for potential variables included in the model based on literature. Moreover, our models had a relatively low pseudo-R-squared. This implies that other unmeasured factors may be associated with knowledge and perception of telepharmacy services among pharmacy students to varying degrees.

Our findings indicate a gap between the knowledge expected to implement telepharmacy services and the willingness of pharmacy students to provide such services in Indonesia. Previous findings that the general population in Indonesia had a positive perception and were willing to use telepharmacy services [47] are an opportunity for pharmacy students. The education system in Indonesia must enhance its responsiveness and capacity to respond to current needs. As such, efforts should be directed toward capacity building for pharmacy students. Specifically, telepharmacy practice models must be included as a subject course in the curriculum, better preparing future pharmacists to perform their roles effectively. Future studies can be conducted with faculty and staff for the assessment of their perceptions on the feasibility to include tele-education into the pharmacy curricula.

## Conclusion

Despite the limited knowledge of telepharmacy, the majority of pharmacy students reported a positive perception and willingness to provide telepharmacy services in their future careers. Student-specific factors such as age and expertise in smartphone usage that associated with knowledge and gender that associated with perception should be considered to facilitate telepharmacy adoption in Indonesia.

### Electronic supplementary material

Below is the link to the electronic supplementary material.

Supplementary Material 1

## Data Availability

The datasets used and/or analysed during the current study available from the corresponding author on reasonable request.
